# Wild-Type Scandinavian Planarian-Derived Extracellular Vesicles Accelerate Skin Wound Healing in Burn and Mechanical Injuries

**DOI:** 10.1021/acsomega.5c11592

**Published:** 2026-03-20

**Authors:** Rakel Bjurling, Hanna Végh, Crispin Hetherington, JinSuck Yang, Roger Olsson, Martin Hjort

**Affiliations:** † Chemical Biology & Therapeutics, Department of Experimental Medical Science, 5193 Lund University, 221 00 Lund, Sweden; ‡ National Center for High Resolution Electron Microscopy, Centre for Analysis and Synthesis, 5193 Lund University, Box 124, Lund SE-22100, Sweden; § Gewissen Co. Ltd, 302 Gate4 Electroland New BLDG 3F. 74, Cheongpa-ro, Yongsan-gu, 04372 Seoul, Korea

## Abstract

Skin wounds remain a clinical challenge, especially for burns and chronic wounds, and existing therapies seldom re-engage the rapid, scar-sparing repair programs observed in nature. Planarians are super-regenerators capable of rebuilding the entire organism from small fragments, and their extracellular vesicles might encode potent prorepair cues. But whether planarian-derived extracellular vesicles (EVs) can enhance mammalian skin healing is unknown. Therefore, we isolated EVs from a wild-type planarian flatworm collected in Sweden and evaluated their therapeutic activity in complementary wound models: a chicken chorioallantoic membrane assay and a human 3D skin model. In our models, planarian EVs significantly accelerated tissue regeneration and wound closure, and improved re-epithelialization and barrier integrity compared to controls. These data indicate that cross-species (xenogeneic) EVs from planarians carry bioactive factors capable of expediting cutaneous repair. Together, the results position planarian-derived EVs as a potential cell-free therapeutic strategy for burns and chronic wounds, motivating additional mechanistic and translational studies for clinical use.

## Graphical abstract

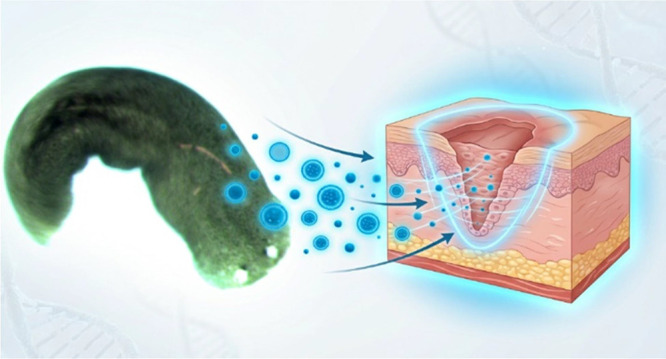

## Introduction

Chronic and acute skin injuries, such as burns, ulcers, and other wounds, pose significant clinical challenges.^1^ Nonhealing wounds affect millions of people worldwide and have become an important medical and socioeconomic burden, with current treatments often inadequate.^2^ Traditional cell-based therapies (e.g., grafting stem cells or skin cells) can aid healing but face severe limitations, including immune rejection, tumorigenic potential, and difficulties in scaling up cell supply.^3^ Consequently, there is growing interest in cell-free regenerative approaches, particularly the use of extracellular vesicles (EVs), to overcome these hurdles.^4,5^

EVs such as exosomes are nanoscale, lipid bilayer-enclosed vesicles released by cells, carrying diverse bioactive cargo (e.g., proteins, lipids, and RNAs), acting to mediate intercellular communication.^6,7^ They offer advantages as therapeutics due to their low immunogenicity, negligible toxicity, and the capacity for engineering or combinatorial delivery with biomaterials.^5,8^ Indeed, during wound healing, EVs are endogenously released from various cells (immune cells, keratinocytes, fibroblasts, platelets) and actively participate in the repair process.^9^ A rapidly growing body of evidence confirms that EVs can modulate key processes in tissue regeneration, reducing injury-induced damage, orchestrating inflammation resolution, and promoting cell proliferation, migration, and angiogenesis necessary for healing. Notably, mesenchymal stromal cell-derived EVs have demonstrated significant wound-healing benefits in preclinical models, underscoring the therapeutic potential of EV-based treatments in dermatology.^9^

While most EV research for wound repair has focused on human or mammalian cell sources, far less is known about EVs from organisms with extraordinary regenerative capacity. Planarians, freshwater flatworms famous for their ability to regenerate entire organisms from small parts, present a compelling model in this context.^10^ Although EVs are known to promote tissue repair in mammals, their roles in highly regenerative animals remain poorly understood. Planarian flatworms can regrow their entire bodies thanks to pluripotent somatic stem cells called neoblasts, which proliferate in response to injury.^11^ It stands to reason that these organisms may leverage EV-mediated signaling during their regeneration. Recent work in the model planarian *Schmidtea mediterranea* has shown that EVs produced by regenerating planarian tissues carry conserved biogenesis markers and can stimulate stem cell proliferation in vivo.^11^ Injecting EVs from regenerating fragments into planarians enhanced the expression of proliferation-related genes and increased neoblast numbers by 50%, demonstrating that planarian EVs contain factors that promote allogenic tissue regeneration. Additionally, a very recent study by Sasidharan showed that planarian EVs contain small RNAs important for cell–cell signaling within the planarians.^12^ These findings open up the intriguing possibility that EVs from highly regenerative species could be harnessed to improve healing in other organisms, such as humans. However, to date, there has been no exploration using invertebrate-derived EVs for human tissue repair, and a significant knowledge gap remains in identifying pro-regenerative structures from such species.

In this study, we address these gaps by investigating EVs from a wild-type planarian species (isolated from a park in Malmö, Sweden) as a potential therapeutic for skin wound healing. We confirmed the regenerative capacity of the flatworm and the ability for long-term culturing in a laboratory environment. We isolated and characterized the planarian-derived EVs using biophysical techniques, confirming their size and morphology. We then evaluated their regenerative efficacy in two complementary models: the chicken chorioallantoic membrane (CAM) model, which provides a highly vascularized in vivo-like platform for wound healing, and a human 3D skin equivalent model that closely mimics human epidermal wound responses. Importantly, we tested the EV therapy on both mechanical wounds and thermal burn injuries to assess the breadth of its effectiveness.

We found that treatment with planarian EVs significantly accelerated wound closure and faster re-epithelization in both models compared to controls. Additionally, when EVs were added to the culture medium, the fibroblast rich bottom layer of the skin samples proliferated at a higher rate, thereby highlighting their potential use as an intervention for age-induced skin thinning.^13^ These results, to our knowledge, represent the first demonstration of an EV-based intervention derived from a highly regenerative invertebrate applied to mammalian wound models. By leveraging the innate regenerative capabilities of planarians, this work highlights a novel strategy for enhancing skin repair. The demonstrated pro-healing effects of planarian EVs underscore their potential as a cell-free therapeutic in regenerative medicine and dermatological wound care, and justify further exploration into the specific molecular cargo and mechanisms by which these vesicles orchestrate accelerated healing.

## Materials and Methods

### Montjuïc Water

Montjuïc water was prepared by dissolving 1.6 mM NaCl, 1.0 mM CaCl2, 0.1 mM MgCl2,1.0 mM MgSO4, 0.1 mM KCI, and 1.2 mM NaHCO3 in Milli-Q water.

### Capturing Planarians and Husbandry

Wild type planarians were caught in Pildammsparken, Malmö, Sweden, using acrylic planarian traps (ebishop.se) placed among decomposing leaves around the pond. The annual temperature range in Malmö is moderate, with mild winters and cool to warm summers. The mean annual temperature is about 9 °C, reflecting the moderating influence of the surrounding seas. Malmö has an oceanic climate (Cfb) according to the Köppen–Geiger classification. The traps were baited with small pieces of raw chicken meat and collected after 24h. The worms were microscopically identified, washed, and placed in EasYFlask Cell Culture Flasks with Montjuïc water (1 mL/worm). The flatworms were maintained at room temperature (18–22 °C), with about 80 worms per T225 flask.

Newly caught planarias were larger and required more water. Experimentally, we found that about 45 worms per flask was suitable for the freshly caught ones. If cultured at too high a density, the planarians disintegrated.

The buffer was replaced at one and 3 days after feeding to remove excess debris. If the culture medium was dirty, additional replacements were done. During buffer replacement, the planarians were also rinsed with 30 mL Montjuïc water to clean them.

The planarians were fed every 7 days with a pea-sized piece of egg yolk or raw chicken liver. After feeding, the planarians moved away from the food and had a visible color change. Planarians were multiplied by cutting them along the anterior-posterior axis into 2 or 3 pieces, depending on size. After feeding, planarians were starved for 7days before being processed for EVs.

### Cell Dissociation

5–7 Planarians were moved to a Petri dish with a pipet and rinsed with Montjuïc water. The planarians were cut several times along the anterior-posterior axis with a razor blade, then transferred with a pipet to a microcentrifuge tube along with 1 mL Montjuïc water with 1 mg Collagenase Type 1 (Gibco) to aid the cell dissociation process. The solution was gently pipetted up and down, 30 strokes over 60 min, with manual rocking between titrations.

The solution was centrifuged at 300*g* for 5 min, supernatant was removed and pellet resuspended in PBS and passed through a 70 μm nylon Cell Strainer (Falcon) to achieve a cell suspension.

### EV Purification

Freshly made cell suspension was centrifuged at 300*g* for 5 min and the supernatant was transferred to a new microcentrifuge tube, and centrifuged at 3000*g* for 30 min. The supernatant was transferred to a new tube, then spun again at 10,000*g* for 30 min.

The supernatant was mixed with an equal volume of 0.22 μm-filtered 10% Poly ethylenglykol 10,000 (#102773909 Sigma-Aldrich) diluted in PBS to aid in EV aggregation. The mixture was incubated at 4 °C for 1 h and then centrifuged at 3000*g* for 30 min to pellet EVs. Supernatant was removed and the pellet containing aggregated EVs was resuspended in 0,5 mL Montjuïc water. Lastly, the EVs were sterile filtered with a 0.20 μm syringe filter (Fisher brand) before use.

### Dynamic Light Scattering

A ZetaSizer nano ZS (Malvern Instruments Ltd.) was used to measure the hydrodynamic radius of the EVs dispersed in Montjuïc water at room temperature. 1 mL EV solution was loaded in PMMA cuvettes for analysis. Following DLS, the EV solution was pipetted into an Eppendorf tube for continued storage or for use in functional assays.

### Fluorescence Microscopy

EVs were stained with the membrane intercalating BioTracker 490 Green cytoplasmic Membrane dye (Merck KGaA) and observed in an Olympus CKX41 microscope.

### Cryo Transmission Electron Microscopy

The EV microstructure was examined using a JEM-2200FS transmission electron microscope (JEOL) specially optimized for cryo-TEM at the National Center for High Resolution Electron Microscopy (nCHREM) at Lund University. It is equipped with a Schottky field-emission electron source and operated at an acceleration voltage of 200 kV. An in-column energy (omega) filter and a 25 eV slit were used. The images were recorded via SerialEM software under low-dose conditions onto a bottom-mounted TemCam-F416 camera (TVIPS). Each sample was prepared using an automatic plunge-freezer system (Leica EM GP) with the environmental chamber held at 20 °C and 90% relative humidity. A droplet (4 μL) taken from a sample was deposited on a lacey Formvar carbon-coated grid (Ted Pella) and was blotted with filter paper to remove excess fluid. The grid was then plunged into liquid ethane (around – 184 °C) to ensure the rapid vitrification of the sample in its native state. Prior to the cryo-TEM measurements, the specimens were stored in liquid nitrogen (−196 °C) before imaging under the microscope using a cryotransfer tomography holder (Fischione Model 2550).

### Chicken CAM Wound Healing Assay

Fertilized eggs from domestic chickens ( *Gallus gallus* ) were washed with 70% ethanol and incubated at 37.5 °C and 60% humidity. The eggs were turned for 30 s 12 times daily in an egg incubator (Chicti). On embryonic development day (EDD) 4, 4–6 mL of albumin was withdrawn through the shell on the side of each egg using a 20 G syringe. The eggshell on the blunt side was removed using a stainless-steel egg topper and a small scalpel. The opening was covered with a 35 mm Petri dish lid, and the egg was placed in an incubator (Heka-Brutgeräte) at 37.5 °C and 60% humidity until used. On EDD 6 the eggs were removed from the incubator and a wound was induced in the chorioallantoic membrane (CAM) by pressing a 100 °C metal rod onto it. The wound could easily be identified from the vascular damage induced. A plastic ring was added around the damage for easy visualization and to make sure EVs stay in place. 30 μL EV solution or PBS was carefully pipetted onto the wound region whereafter the eggs were put back into the incubator. 24h later, samples were imaged and the wound diameter registered.

### Artificial Skin Wound Healing

Full thickness artificial skin models (EpiDerm FT, Mattek Corporation) was used to evaluate the EVs’ ability to promote wound healing and skin growth. Upon arrival, the hanging inserts holding the tissue samples were moved to new 6-well plates for continued culture. Two wells were cultured in 2.5 mL EpiDerm medium­(Mattek), 2 were cultured in 2.25 mL EpiDerm medium +250 μL EVs, and 2 were cultured in 2.5 mL EpiDerm medium but with 30 μL EVs added on top of the tissue. Skin wounds were introduced in a separate set of samples using a 3 mm biopsy punch. The wounded samples were treated similar to the intact ones. Every day, the medium was exchanged, and fresh EVs were added.

After 3days in culture, the samples were fixed in 4%PFA overnight, embedded in paraffin, sectioned, and stained using hematoxylin and eosin staining. Sections were imaged using an Olympus microscope and skin layer thickness measured.

### Immunogenicity Experiments

Human primary peripheral blood mononuclear cells (PBMCs) were isolated from primary blood obtained from healthy donors at Lund University Hospital, Lund, Sweden. PBMCs were separated by Lymphoprep (Stemcell technologies) density gradient centrifugation. PBMCs were cultured in Roswell Park Memorial Insitute (RPMI 1640, Gibco) medium supplemented with 10% fetal bovine serum (FBS, Gibco) and 1% pencillin-streptomyocin (Gibco) in a humidified incubator at 37 °C and 5% CO_2_.

PBMCs were analyzed using flow cytometry. Cell viability was evaluated using Calcein AM as viability stain. For flow cytometry, cells were resuspended in FACS buffer (10% FBS, 2.5 mM EDTA, 0.05% Sodium azide in PBS). Data was acquired using BD Symphony A1 flow cytometer (BD Biosciences) and analyzed by FlowJo software (v10.10.0, FlowJo LLC, BD Biosciences). To measure the cell count, 50 μL of each sample was aspirated and live cell count was quantified. Gating strategy is shown in Supporting Figure S1 whereas representative flow cytograms are shown in Supporting Figure S2.

### Statistical Testing

Statistical testing was performed using GraphPad prism v10 using unpaired student’s *t* test unless otherwise noted. Significance presented in graphs as * (*p* < 0.05), ** (*p* < 0.01), and *** (*p* < 0.001).

## Results and Discussion

### Planarian Culture and EV Extraction

Wild type planarians were caught in Pildammsparken in Malmö, Sweden, by placing acrylic planarian traps overnight, baited with small pieces of chicken liver. The traps were completely submerged in shallow water close to decomposing leaves to give the highest yield. The planarians were manually sorted and washed before being kept in culture at up to 50 worms per T225 flask at room temperature and in the dark. Too high planarian density in the culture flasks led to the complete disintegration of the worms. The medium, Montjuïc water, was changed at least every 3 days to remove debris or waste being shed from the worms. Once per week, the worms were fed with egg yolk or chicken liver. Successful feeding was observed through a change of planarian color and by their lack of interest in additional food.

To confirm the regenerative capacity of the planarians, they were cut along the anterior-posterior axis. When split in half, Figure 1b, both the head and tail parts retained movement, albeit more active for the head part, see supplemental movie 1. After 7 days, a new head with eyes clearly visible had regenerated on the tail part. After 2 weeks, both segments had formed complete worms. We used this regenerative capacity to expand our cultures in vitro. Each worm was cut 2 or 3 times along the anterior-posterior axis to form new individuals over the coming 1 to 2 weeks.

**Figure 1:**
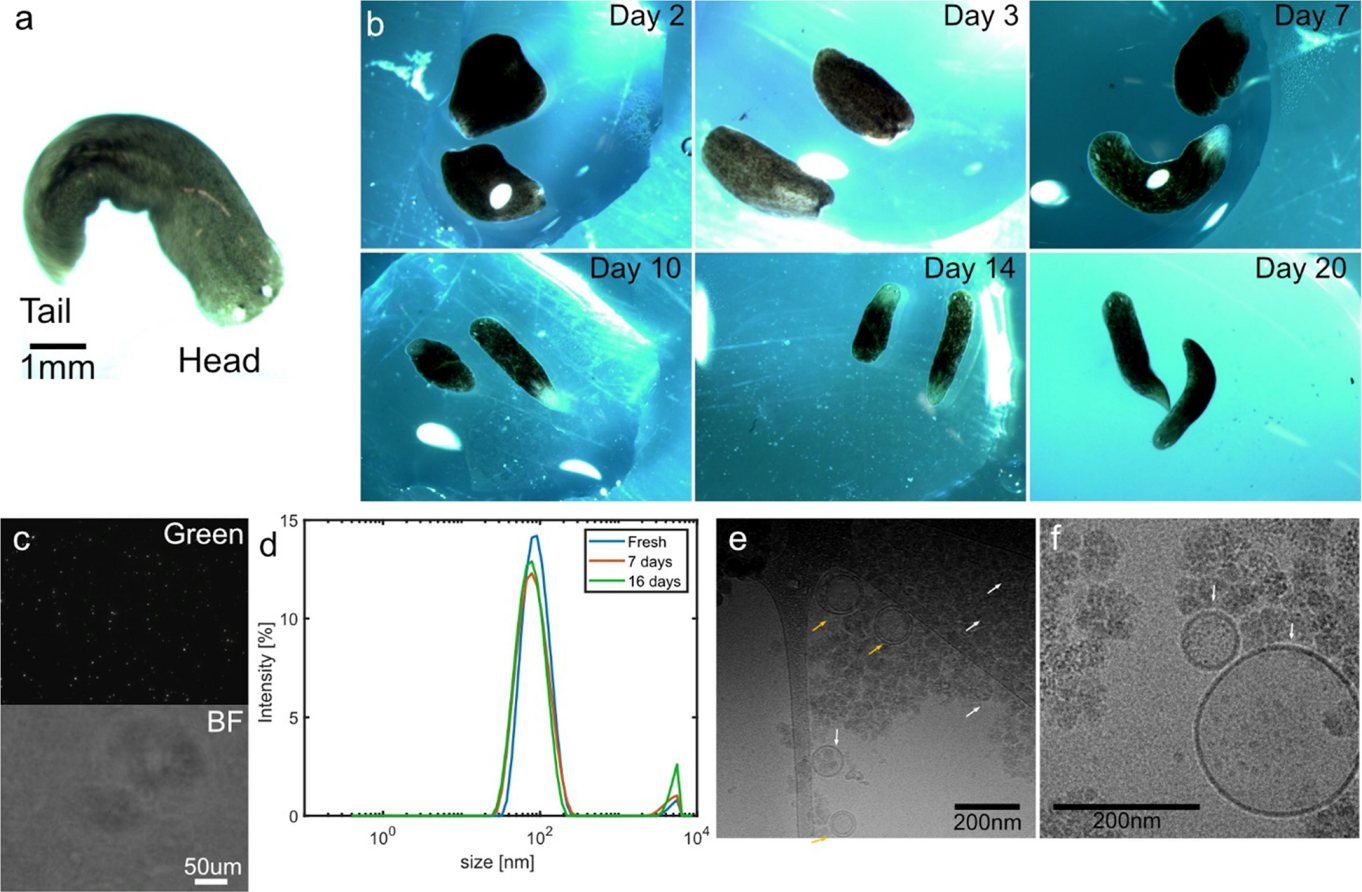
Planarian regeneration, EV extraction and analysis. (a) Representative planarian showing characteristic head and tail region. (b) Micrographs showing the regeneration process after microscopy guided cutting of a planarian. Days after cut as indicated in each picture. (c) Purified EVs stained with BioTracker membrane dye as observed in fluorescence microscopy (green channel) and brightfield (BF). Individual EVs can be seen in the green channel, but are not visible in BF. (d) DLS measurements showing the size range for EVs freshly purified (blue) and stored for 7 days (red) or 16 days (green). All samples had a mean diameter of 90 nm. (e, f) Cryo-TEM showing EVs with either 1 (white arrows) or 2 (orange arrows) lipid membranes. In (f), dark spots can be seen inside the EVs, indicating the existence of encapsulated biomolecules.

EVs were extracted from the planarians following tissue disintegration and a set of centrifugation steps, see the Supporting Information for details. Planarians were starved for 7 days and washed carefully to ensure that the EVs would be coming from the planarian cells only. First, the planarians were cut repeatedly into small pieces, followed by tissue dissociation using collagenase. Following filtration to remove undigested tissue, pelleted cells and larger particles were discarded after centrifugation. The supernatant, containing EVs, could be further processed using advanced techniques such as ultracentrifugation, size-exclusion chromatography, or bespoke microfluidic devices to extract and concentrate the EVs. However, we used polyethylene glycol (PEG) to aggregate the EVs, thereby making it possible to pellet them in conventional table-top centrifuges using moderate speeds. Lastly, the PEG-EV pellet, which is not visible to the naked eye, was resuspended in Montjuïc water, ready to use for downstream processing.

As expected, the EVs were too small to be resolved in conventional optical microscopy. Instead, we added a fluorescent membrane dye that intercalates in lipid membranes and fluoresces green when excited with blue light. The EVs could then be seen as point like bright spots indicating the existence of lipid membrane-enclosed structures without signs of aggregation, Figure 1c. The presence of EVs in the solution is a quick and straightforward step to verify successful purification.

To achieve a more detailed understanding of the EV structure, we turned our attention to cryo transmission electron microscopy (cryo-TEM). The EV sample was pipetted onto a lacey carbon coated TEM-grid, blotted, flash frozen in liquid ethane, and kept at or near liquid nitrogen temperature during transfer to the cryo-TEM. Circular EVs with diameters of about 50–200 nm were found dispersed in the vitreous ice, Figure 1e,f, thereby falling within the exosome size range of 40–200 nm.^14^ A single lipid bilayer enclosed some of the EVs, whereas some had 2 bilayers. Multibilayer EVs have also previously been observed and showcase the strengths of using cryo-TEM to image the sample directly.^15^ Interestingly, no EVs with more than two bilayers were observed. Inside the EVs, electron-dense regions acting to scatter the illuminating electron beam could be observed (Supporting Figure S3), corroborating the hypothesis that they contain bioactive molecules. Around most of the EVs, disordered structures were also observed, presumably being PEG residues combined with salts from the Montjuïc water.

Fluorescence microscopy and cryo-TEM confirmed discrete EVs, and dynamic light scattering (DLS) provided a statistically meaningful size-distribution profile. DLS is a nondestructive method utilizing the scattered intensity of a laser beam passing through the sample, making it possible to estimate the EV size distribution while still being able to use the sample for downstream experiments.

Freshly purified EVs showed a single peak in DLS extending from 30–200 nm with a mean hydrodynamic particle size of 90 nm, Figure 1d. These results confirm the TEM findings, which showed EVs of the same diameters. Notably, no peak was detected in the single-digit-nanometer range, consistent with the absence of freely dispersed proteins. A minor μm-scale population was observed; while its origin is uncertain, it may reflect PEG-associated aggregates, consistent with TEM. EVs harvested from the supernatant of cultured planarian cells (rather than directly from the worms) showed larger EVs as well as signatures of free protein in solution (Supporting Figure S4). However, no EV sample showed any quantifiable amounts of free protein when measured using a Bradford assay (Supporting Figure S5). All experiments below are performed using EVs derived from the worms, and not using cell supernatant.

The EVs were stored at 4 °C in Montjuïc water and reanalyzed 7 days and 16 days later (the longest time-point measured) without any notable change in appearance as observed in DLS. Surprisingly, a minor shift toward a smaller EV diameter was observed. Hence, even without added stabilizers, the EVs are long-term stable and not prone to either aggregating, fusing, or dissolving.

### Burn Wound Healing

Having established a methodology to generate, purify, and analyze EVs, we turned our attention to using them for wound healing studies. The chicken chorioallantoic membrane (CAM) is a highly vascularized, extra-embryonic tissue that forms in ovo around 3 days after fertilization. The CAM has recently been used as a robust and ethically justifiable way to generate burn wounds, which removes a lot of the variability associated with other assays.^16^ By gently pushing a metal rod heated to 100 °C into the CAM, shallow vasculature breaks without causing major bleeding. The damaged region can be identified optically (green dashed circle in Figure 2a) and its extent over time can be quantified to monitor wound healing. After damage induction, wounds with a diameter of 2.5–4 mm were seen where variations in rod placement contributed to the variability.

**Figure 2:**
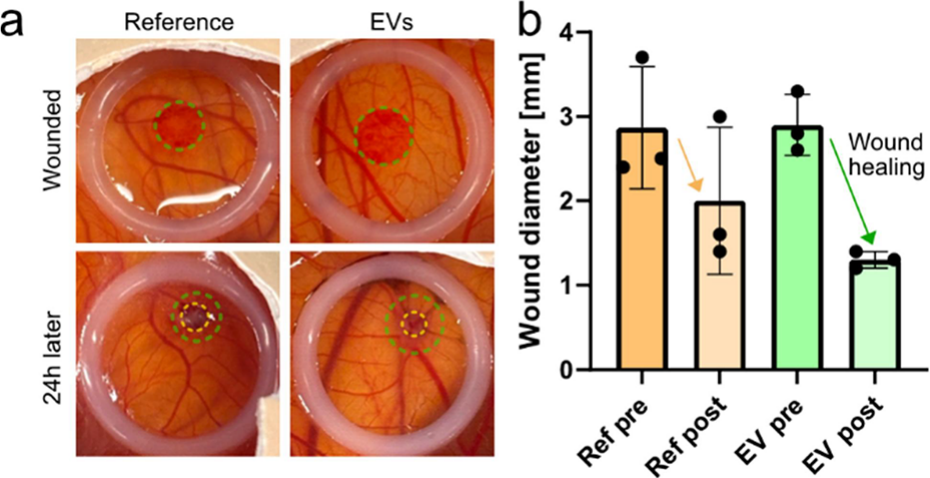
Burn wound in a chicken embryo chorioallantoic membrane. (a) Eggs with CAM burn wounds before (green dashed circle) and after treatment (yellow dashed circle). A 1 cm white plastic ring was used to mark the region of interest in the CAM. (b) Graph summarizing the wound diameter. Reference denotes treatment with PBS. Three eggs were used for each condition. Stars denote statistical significance: * *p* < 0.05.

Planarian EVs added to the burn wound resulted in faster healing. Immediately after wounding the CAM, 30 μL phosphate buffered saline (PBS) or EV solution was added. A plastic ring was placed around the wound to ensure that the liquids would stay in place and to aid in finding the wound. The eggs with PBS or EVs were placed in an egg incubator and kept for 24h before remeasuring the wound diameter (dashed yellow circles).

All samples displayed wound healing (i.e., smaller diameter wounds), but it was more efficient when EVs were added. The wound color changed from bright red to a darker red. Initially, the average wound diameter was the same for the two cohorts. After treatment, the average EV wounds were not only smaller, but also displayed a narrower size distribution. After 2 days, the wounds were typically fully healed. Taken together, the experiments show that the EVs are biologically active and can be used for a more rapid burn wound healing in the CAM model.

### Human Artificial Skin Regeneration

Encouraged by the results in the CAM model, we explored whether the planarian EVs can also be used on artificial human skin samples (Epiderm EFT-412, MatTek). These commercially available full thickness skin samples come with a fibroblast rich bottom layer, cell and collagen rich dermis, epidermis, and top stratum corneum. Their close resemblance to primary skin samples makes them an excellent test bed for skin regeneration research.^17,18^ It aligns well with recent FDA initiatives to minimize the use of animal models.^19^

The samples were kept in an incubator at an air–liquid interface with daily medium changes and EV addition either on top of the stratum or into the medium. After 3 days in culture, the samples were fixed in PFA for 1 day, embedded in paraffin, sectioned, stained with hematoxylin and eosin (H&E), and analyzed using a microscope.

All samples showed the expected layered morphology with no major difference in cell morphology. The layering was easily observed in the stained sections, as indicated in Figure 3, and we measured the thickness of the different layers to quantify the effect of the EVs. No significant differences were observed in the upper layers of the skin, possibly related to poor penetrance of the EVs through the stratum.^20^ However, in the bottom fibroblast rich layer, having EVs in the culture medium resulted in a significant increase in thickness. This is interesting from a skin regeneration point-of-view since fibroblasts are the primary cell type responsible for generating collagen, the most abundant extra cellular matrix protein, essential for maintaining skin elasticity and strength.^21^ More fibroblasts therefore means the possibility to produce more extracellular matrix over time, enabling skin rejuvenation and combating age-induced skin thinning.

**Figure 3:**
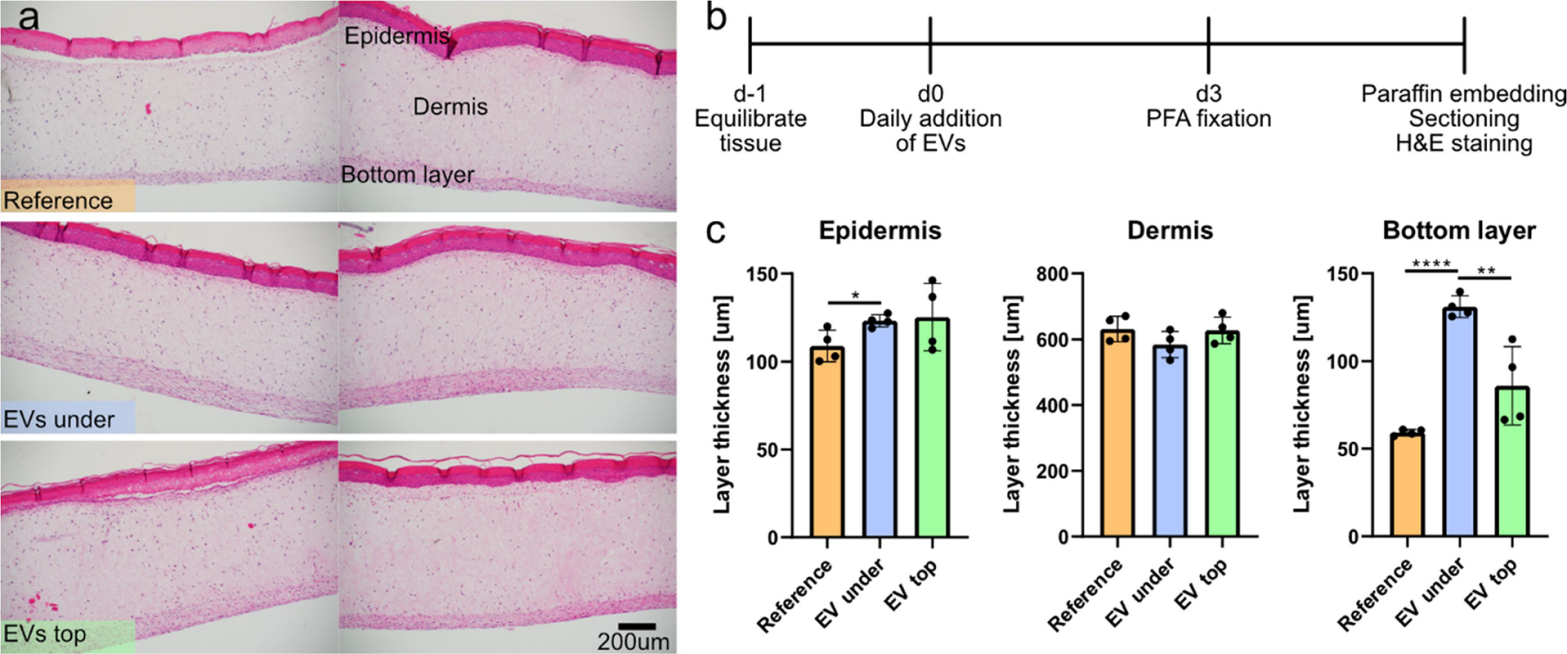
Pristine human artificial skin samples. (a) Optical micrographs showing H&E stained sections with the different layers indicated. The skin samples were cultured as recommended (“reference”), with EVs added to the culture medium (“under”) or with EVs on top of the epidermis (“top”). (b) Timeline depicting the outline of the experiment. (c) Thickness of the different layers. Two biological and two technical replicates per condition. Stars denote statistical significance: * *p* = 0.026, **** *p* < 0.0001, ** *p* = 0.0081.

### Puncture Wound Healing in Human Skin Assay

The artificial skin can also be used to evaluate puncture wound healing. A three mm biopsy punch was used to generate holes throughout the center of the skin samples. During a three-day culture, EVs were added either into the medium (“under”) or topologically on top of the skin (“top”) at the wound area, Figure 4. The medium was exchanged daily, and EVs were refreshed. After sectioning and H&E staining similar to the pristine samples, it was possible to determine how far into the wound region the epidermis had regrown. This is a direct measurement of the wound healing process.^22^ The samples with EVs dosed on top resulted in a more efficient wound healing, extending on average 400 μm into the wound, compared to either reference (230 μm) or EVs in medium (240 μm). The effect seen when dosing the top EVs could be related to a locally higher concentration, or a different cellular uptake pathway where damaged skin is more efficient in taking up the EVs. The increased proliferation and migratory capacity of the skin cells is exciting and has also very recently been reported when dosing human EVs.^23^ Topologically applied EVs can therefore be used to promote wound healing also in human derived models.

**Figure 4:**
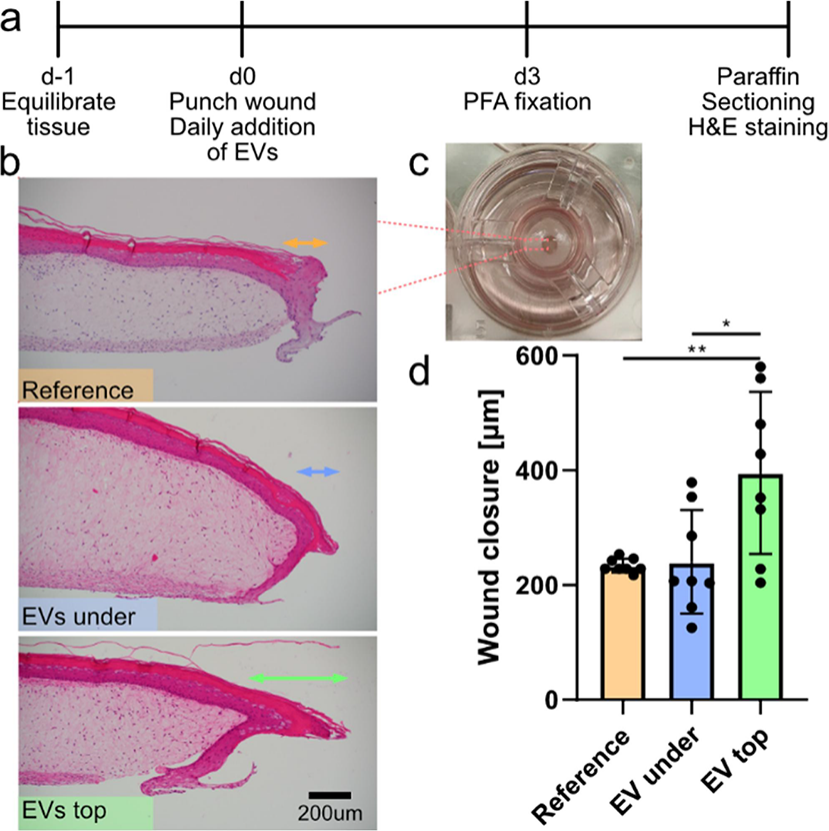
Puncture wound healing in a human skin assay. (a) Timeline of the experiment. (b) Representative micrographs depicting epidermis growth into the wound region (right-hand side). Double arrowed lines depict extent of the outgrowth. (c) Photograph showing a well-insert with a punctured skin sample in the middle. (d) Graph summarizing the wound closure (orange is reference, blue is with EVs in medium, green is EVs on top). Two biological replicates and two sections for each condition. Stars denote statistical significance: Top vs ref *p* = 0.0063, top vs under *p* = 0.020.

Even though the skin is punctured through, the EVs added on top will not immediately be diluted into the medium since it sits on a porous membrane support limiting diffusion. However, we do expect some diffusion of the EVs, which might explain the larger variability for the EV samples compared to the reference. Interestingly, for the wounded samples, EVs dosed underneath also seemed to increase the bottom layer thickness, as was observed for the intact skin samples.

### Human Primary Immune Cells

Exposing human primary immune cells to planarian EVs provides insight into their immunogenicity. Fresh human primary peripheral blood mononuclear cells (PBMCs) were purified from blood using lymphoprep gradient centrifugation to separate the buffy coat containing lymphocytes, monocytes, and granulocytes. The cells were cultured in a 6-well plate at a density of 1.5 M cells in 2 mL RPMI medium per well. The RPMI medium is optimized to maintain the cells in a healthy and fit state. Hence, it is expected that cell viability decreases when the medium is diluted with hypotonic Montjuïc water. Cells were cultured in medium supplemented with either 25% sterile filtered Montjuïc buffer or 25% EV solution. Reference cells were cultured in 100% RPMI. With this experimental design, the setup allows for differentiation between EVs and buffer-induced effects.

After 3 days in culture, the cells were imaged and analyzed using flow cytometry to quantify cell numbers and cell viability. In the imaging, no major differences were observed between the samples apart from more elongated cells showing up in the reference sample (medium), presumably being monocyte/macrophage related, Figure 5. To count cells using flow cytometry, the same volume (50 μL) was aspirated from each sample, and the number of viable cells was counted. We observed a slight drop in the samples with diluted medium, likely related to the resulting lower ionic strength of the medium. Importantly, the cells exposed to EVs showed similar cell numbers as those supplemented with Montjuïc water alone. No significant differences in cell number, size, or activity were observed under this simplified in vitro condition, but the immunogenicity still requires subsequent systematic evaluation. We do note that human derived EVs have previously been found to be nonimmunogenic,^24^ which is important since inflammation is a core part in extra cellular matrix degradation in the skin.^25^ Recently, a study on coriander derived EVs also showed good biocompatibility when used in an in vivo mouse setting.^26^ We also mapped the cell viability in the PBMCs as evaluated using the Calcein AM viability stain. Both the Montjuïc and the EV group showed slightly lower cell viability compared to the reference cells as expected when cultured in dilute media.

**Figure 5:**
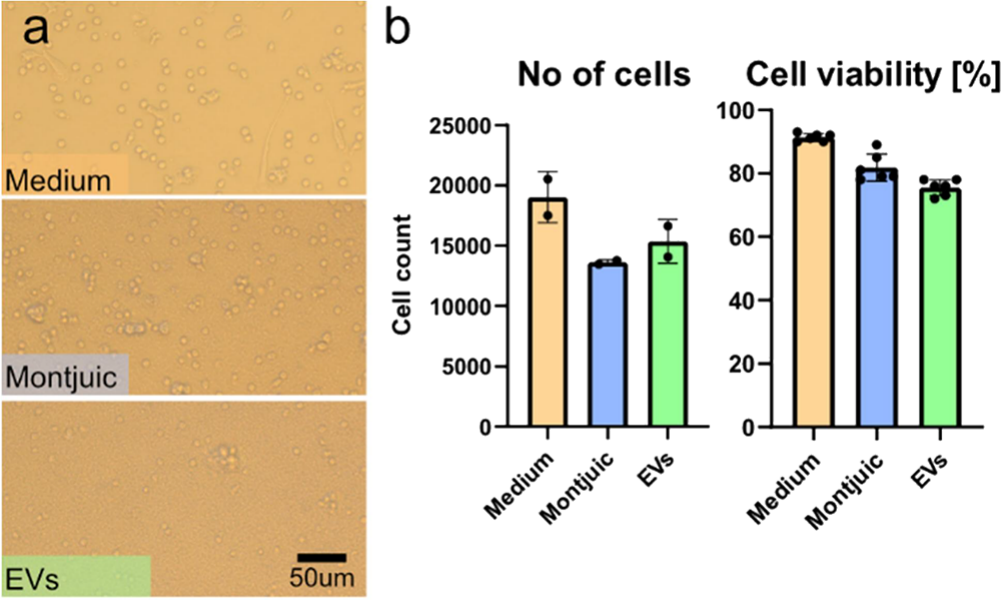
Human PBMCs cultured with EVs. (a) Optical micrographs depicting PBMCs after 3 days culture in medium, medium and buffer (Montjuïc), or medium and EVs (EV). (b) Graphs showing cell count and cell viability of the PBMC after 3 day culture, as measured by flow cytometry. Calcein AM was used as a viability stain.

## Conclusions

Herein, we have shown that EVs from regenerating wild-type planarians promote wound healing and assist in skin regeneration. Planarians were caught in southern Sweden and their regenerative ability was confirmed by dividing them and allowing them to form new worms. EVs were extracted by dissection and tissue digestion. Subsequently, their purification was enabled by PEG aggregation and table-top centrifugation. Cryo-TEM revealed EVs enclosed by one or two lipid bilayers with electron-dense regions inside, presumably harboring biomolecules. The EVs were found to be long-term stable in buffer without any added stabilizers.

EVs dosed on burn and puncture wound models accelerated the healing process thereby showing a cross-species functionality. Interestingly, human skin model samples with EVs added to the cell medium presented a thicker fibroblast rich bottom layer already after a few days in culture. The nonhuman EV origin provides a potential benefit in reducing risk of pathogen transmission.^27^ Further studies should evaluate if the thicker layer makes the EVs an alternative to combat age related skin thinning.^28^

In conclusion, we have presented a hypothesis-generating methodology to extract EVs from wild type planarians and their use for wound healing by showing upregulated fibroblast regeneration as well as increased keratinocyte migration and proliferation in our models. This research paves the way for further studies in cross-species skin healing and rejuvenation to detail mechanism of action as well as any potential immunogenicity.

## Supplementary Material





## Notes

Supplemental Figures S1–S5 showing gating strategy, representative flow cytograms, and additional cryo-TEM images (PDF)
(Movie S1) Movie depicting a planarian being split along the anterior-posterior axis, followed by regeneration over 20 days (MP4)
