# Supplementary material for: Wild-Type Scandinavian Planarian-Derived Extracellular Vesicles Accelerate Skin Wound Healing in Burn and Mechanical Injuries
Source: ACS Omega. 2026 Mar 20;11(13):20683–90. doi: 10.1021/acsomega.5c11592 (PMC13063049; doi:10.1021/acsomega.5c11592)
Supplement: Supplementary file 1 [file ao5c11592_si_001.pdf]

Supporting information for:

Wild-type Scandinavian Planarian-Derived Extracellular Vesicles Accelerate Skin Wound Healing in Burn and Mechanical Injuries

Authors: Rakel Bjurling[1], Hanna Vegh[1], Crispin Hetherington[2], JinSuck Yang[3], Roger Olsson[1], and Martin Hjort[1*]

Author Affiliations:
[1] Chemical Biology & Therapeutics, Department of Experimental Medical Science, Lund University, 221 00 Lund, Sweden
[2] National Center for High Resolution Electron Microscopy, Centre for Analysis and Synthesis, Lund University, Box 124, Lund SE-22100, Sweden
[3] Gewissen Co. Ltd, 302 Gate4 Electroland New BLDG 3F. 74, Cheongpa-ro, Yongsan-gu, Seoul, Korea
[*] Corresponding author email: martin.hjort@med.lu.se

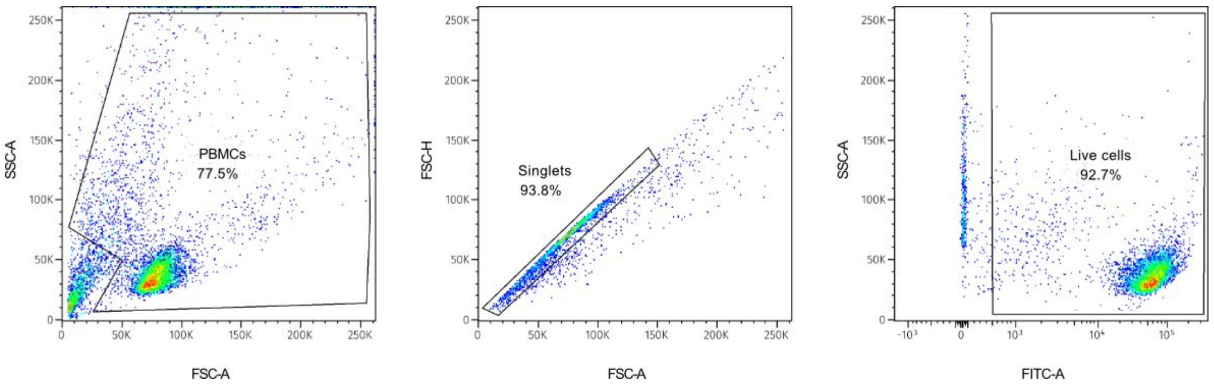

Figure S1. Gating strategy for the PBMC flow cytometry. Representative sample cultured in medium.

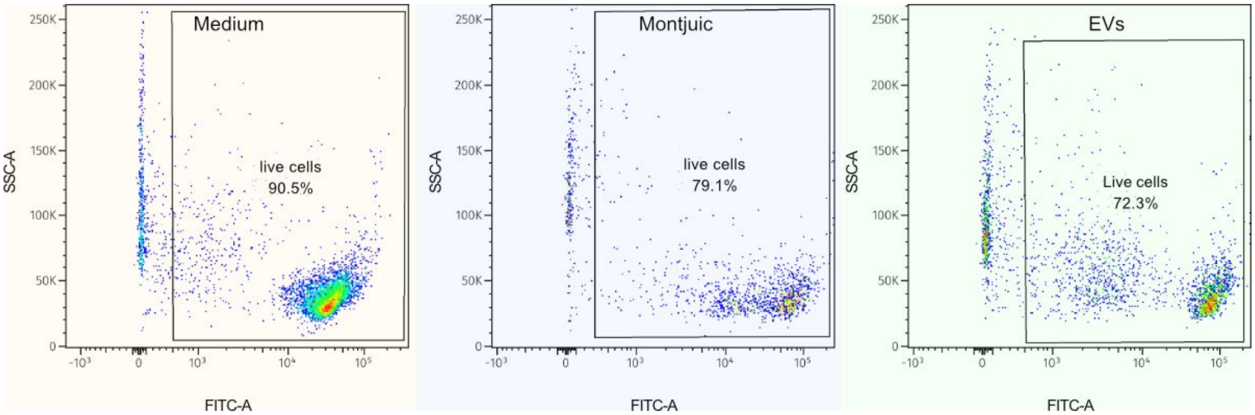

Figure S2. Representative flow cytograms showing the live cell population for PBMCs in the three different culturing conditions, as marked in the figure.

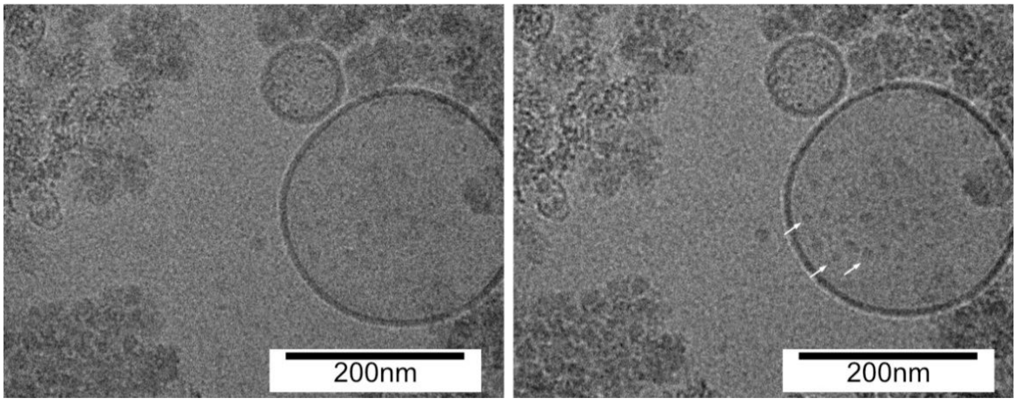

Figure S3. Cryo-TEM images obtained at different foci. White arrows point to electron dense regions inside the EV.

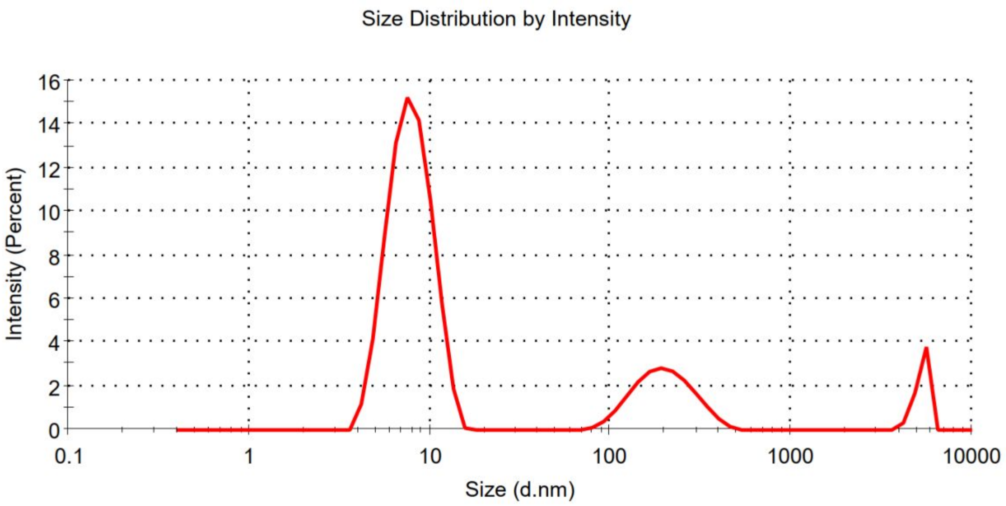

Figure S4. DLS size distribution obtained when analyzing EVs *purified from the supernatant of cultured planarian cells* dissociated from the worms. Please note the existence of a sub 10nm peak not appearing when purifying EVs directly from the worms.

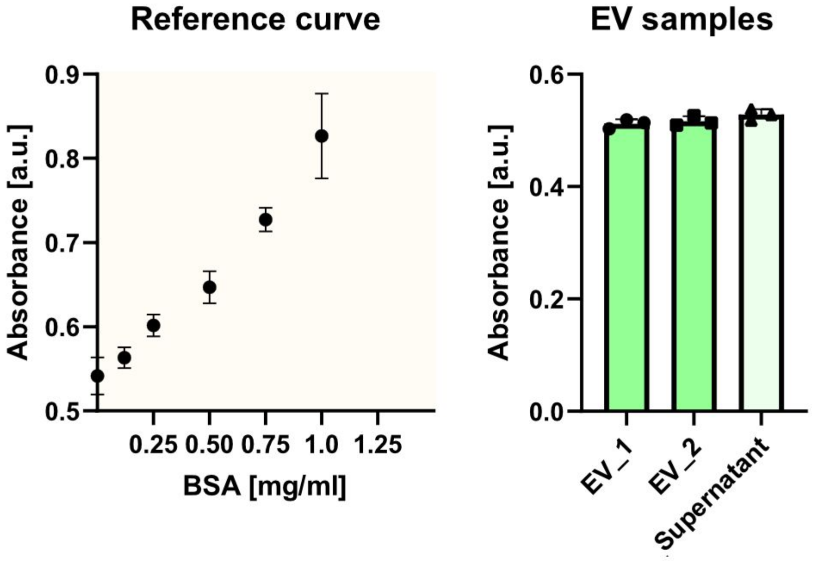

Figure S5. Quantifying protein content outside the EVs in a Bradford assay (Sigma Aldrich B6919). Absorbance of the Bradford reagent at 595nm was obtained on a microplate reader (Spark Cyto, Tecan) and standardized using Bovine serum albumin (BSA, Sigma Aldrich #A4503) reference samples (n=3). The protein concentration in two separate EV samples, “EV_1” and “EV_2”, was under the detection limit of the assay (1ug/ul), 3 technical replicates. EVs purified from planarian cells in solution, “Supernatant”, (rather than directly from the worms), also showed protein concentration under the detection limit, 3 technical replicates.
